# Neuroscientific and neuroanthropological perspectives in music therapy research and practice with patients with disorders of consciousness

**DOI:** 10.3389/fnins.2015.00273

**Published:** 2015-08-04

**Authors:** Julia Vogl, Astrid M. Heine, Nikolaus Steinhoff, Konrad Weiss, Gerhard Tucek

**Affiliations:** ^1^Department of Social and Cultural Anthropology, University of ViennaVienna, Austria; ^2^Department of Music Therapy, IMC University of Applied SciencesKrems, Austria; ^3^OptimaMed Neurological RehabilitationKittsee, Austria; ^4^Department of Neurology, Regional Hospital HocheggGrimmenstein, Austria; ^5^Department of Nuclear Medicine, Regional Hospital Wiener NeustadtWiener Neustadt, Austria

**Keywords:** music therapy, neuroanthropology, disorders of consciousness (DOC), unresponsive wakefulness syndrome (UWS), Positron Emission Tomography (PET)

## Abstract

A growing understanding of music therapy with patients with disorders of consciousness (DOC) has developed from observing behavioral changes and using these to gain new ways of experiencing this research environment and setting. Neuroscience provides further insight into the effects of music therapy; however, various studies with similar protocols show different results. The neuroanthropological approach is informed by anthropological and philosophical frameworks. It puts emphasis on a research *with* and not just *on* human beings concerning the subject/object question within a research process. It examines relational aspects and outcomes in the context of working in an interdisciplinary team. This allows a broader view of music therapy in a reflective process and leads to a careful interpretation of behavioral reactions and imaging results. This article discusses the importance of the neuroanthropological perspective on our way of obtaining knowledge and its influence on therapeutic practice. It is important to consider how knowledge is generated as it influences the results. Data from two cases will be presented to illustrate the neuroanthropological approach by comparing quantitative PET data with qualitative results of video analyses.

## Introduction

A growing understanding of music therapy with patients with disorders of consciousness (DOC) has developed from observing behavioral changes and using these observations to gain new ways of experiencing the research environment and setting. Music therapy has been used to support the neural and behavioral rehabilitation of individuals with unresponsive wakefulness syndrome (UWS) for over 20 years now. An increase in music therapy research points to its importance (Gustorff and Hannich, 2000; O'Kelly et al., 2013; Magee et al., 2014; Raglio et al., 2014; Magee and O'Kelly, 2015) and is additionally supported by research on the neurological impact of music on patients with DOC (Okumura et al., 2014; Verger et al., 2014; Castro et al., 2015).

Where the aim of music therapy is to support the rehabilitation of patients with DOC, studies of behavioral changes are crucial for detecting reactions to music therapy. Still, the simple observation of behavior poses a risk for misinterpretation and misdiagnosis (Andrews et al., 1996; Giacino et al., 2009) because behavioral responses can be limited by cognitive dysfunctions and masked by the inability to execute movement and reactions (Giacino et al., 2009; Fingelkurts et al., 2014; Stender et al., 2014). Neuroscience helps music therapy gain knowledge of the physiological effects of musical elements, which is useful for the theoretical foundation of music therapy. However, images of the brain do not capture the meaning of music therapy for the patients and the impact on their daily life (Nettleton et al., 2014). Although neuroscience is a valuable complement to music therapy research, especially with patients with DOC, it is an illusion that everything can be seen in the brain (Cohn, 2004; Nettleton et al., 2014). It is essential for us to note that we see music therapy as something beyond the simple act of listening to music. It is a complex process built around a therapeutic relationship between the patient and therapist. Therefore, research on music therapy needs a broader approach and interpretation of results, especially when working with patients who cannot communicate their experience verbally, where special attention is required and it is important to find indications for the effect of music therapy.

In an attempt to bridge the gap between research and practice we introduce a neuroanthropological perspective to music therapy research.

## The neuroanthropological approach

The neuroanthropological approach should be seen as a response to challenges occurring within an interdisciplinary research setting, which includes the use of neuroimaging techniques and multidisciplinary methods. Daniel Lende and Greg Downey, who are the key actors within this field, have pointed out a holistic approach, trying to bridge brain and environment, drawing on insights of anthropology and psychology regarding individual-environment interaction (Lende and Downey, 2012). Although it is basically an anthropological approach (Roepstorff and Frith, 2012), we use the prefix “neuro” to emphasize the neuroscientific research, the neurorehabilitation environment and the interdisciplinary approach of our work.

An important, epistemological question, also within disciplinary borders, is how knowledge is generated and, in particular, how this influences the research results. The task was to challenge the research practice itself and simultaneously collect objective data on changes in brain activity.

The field of neuroanthropology is also described as a humanistic science (Domínguez et al., 2009). Within the whole research process each patient is seen as a human subject and not as an object of the study. We want to conduct research *with* the patient and not *on* the patient. This attitude mirrors the approach of music therapy itself, which can be seen as a *relational medicine*, in which the therapist opens up to the individual needs of every patient and tries to meet them at an egalitarian level.

## An anthropological approach to music therapy

Our concept of music therapy (Tucek, 2014; Tucek et al., 2014) derives from an anthropological perspective, which historically developed from the models of traditional oriental music therapy and ethno music therapy to the current model of music therapy in Krems. We are oriented toward an individual, bio-psycho-social approach, asking for individual needs of patients and their personal meaning of music therapy. Inspired by theories of embodiment (Csordas, 2002; Storch and Tschacher, 2014), which describe the engagement of culture and individuals through sensual perception and experience, we believe that the meaning of music in therapy develops within the therapeutic session as a specific tool of communication between the patient and the therapist. In contrast to the theories describing communication as an exchange of certain messages between sender and receiver, we assume that the meaning of communication, as well as of music and music therapy, develops in an interaction between humans and their environment. To paraphrase Simon Rattle (2004), “music is not just what it is, but is that what it means to the people.”

The basic aim of music therapy is to encourage the individual resources of the patients and an allostatic regulation (Schulkin, 2004), helping them regain a mental and physical stability. Therefore, music therapy can be activating on the one hand, e.g., by moving the patient's hand on an instrument or along with a rhythm. On the other hand, it can encourage balance and relaxation by playing improvisations in e.g., playing to the rhythm of the patient's breath. However, as the needs and meaning of interventions differ from person to person, the central concern is to meet and approach each patient individually.

For a positive outcome it is essential to establish a relationship between the patient and the music therapist. Music therapists not only work with musical interventions but also via a human relationship in which music creates a special kind of frame for this intervention. Within this framework music therapy tries to find new ways of connecting with the patient and to establish a communication beyond words; i.e., a way of communicating on a very emotional and relational level which is based on patients' needs and capabilities. From an anthropological perspective, the aim of music therapy is to transform the foreign, clinical environment (*Umwelt, “around-world”*) of patients to their contemporaries (*Mitwelt, “with-world”*) (Binswanger, 1963; Prinds et al., 2013). By addressing the patient individually and opening up to individual needs and reactions, music therapy is formed not only *for* the patient but *with* the patient.

Amidst a hospital setting with technical apparatus and noise, one always runs the risk of losing sight of the human being. According to our understanding of music therapy, as it is applied in Krems, one essential aim of music therapy, especially within intensive care units, is to *humanize*, also in terms of existential orientation. Music therapy tries to build a connection to the patient on an individual, relational level. Being aware of the patient's biography and their new and constantly changing context, the therapist approaches with an inquiring attitude.

It is important for the music therapist to get an understanding of this patient's culture and include it in their therapeutic considerations. In this context, culture is not the idea of “*isolated societies with shared cultural meanings*” but rather one's own “*local world*,” as e.g., the environment of the patient and their family. This culture comprises, among others, the patients' relatives, musical preference, ability to play an instrument and personality, and affects all aspects of their experiences (Kleinman and Benson, 2006). This leads us to the point why anthropology, its core methodology (i.e., ethnography) and its interpersonal, intersubjective nature are important for a music therapist. They help us appreciate and humanly engage with the differentness of a patient, particularly with DOC, and get a deeper understanding of the patient's needs. Nevertheless, we are aware of the fact that, however confident our interpretations might seem to us, they are always limited by the analytic tools and research methods we use and by the epistemological limits of what we actually mean by intersubjectivity (Willen and Seeman, 2012).

Patients with DOC do not only have their “local world,” but they are in a very special situation in a “liminal period” (Turner, 1967) in which familiar norms are absent and received notions of personhood are destabilized (Nettleton et al., 2014). Without the possibility of verbal conversation they have to readapt to a new situation of life by mourning about the loss of their former life and by regaining confidence in their current and future situation. The same holds true for the family members who cannot share their experience with the patient in the way they are used to, and have to go through this reorientation process regarding a life under completely new conditions. The therapist has to be aware of changes caused by this reorientation syndrome (Steinhoff, 2012) and has to consider the individuality of each person involved. In order to connect with a person in such a transition it is important to empathize with the lived human experience of the patient's situation and to approach the patient as an individual human being “facing danger and uncertainty” (Kleinman and Benson, 2006). To help the patient regain certainty and stability, building trust can be seen as a basis for therapeutic relationship. For this, the therapist needs flexibility which is also required in research with human beings.

## Neuroanthropological perspective in music therapy research

When it comes to carrying out research within a therapeutic setting with human interaction as we do in music therapy, many important aspects happen on a subjective level which cannot be sufficiently illustrated in objective terms. Domínguez argues that subjects, subjectivity and understanding are not only absent but obscured and marginalized in favor of the scientific process and its results (Domínguez, 2012), and that subjectivity has a negative attribution *per se*. But why do we need such a radical omission?

Neuroanthropology takes up the idea that knowledge is neither built on objective data nor subjective perception alone (Domínguez, 2012). It does not need an “either/or” nor an “and” but a “with” in terms of interaction between them. The data generated by the PET scanner shows the neural changes during music therapy, but it does not explain the reason for this effect. While it is important to look at this data, an increase in brain activity does not tell us much about the patient's situation. When and how does a patient actually benefit from music therapy and what exactly does he benefit from? To answer these questions we need a more detailed and careful intersubjective interpretation of interactions between the patient and the therapist (Nettleton et al., 2014). Qualitative data collected within the therapeutic setting is essential for our understanding of the effect of music therapy, as it provides more details on it and strengthens the interpretations of the results.

We have tried to jump onto this ontology-epistemology rollercoaster and use insights gained from intersubjective communications and reflective processes to adapt the experimental script design. This helps us to ask novel questions for improving the research process as well as the therapeutic setting.

To give an example, we would like to describe part of our pilot study in which we tried new ways of examining the effect of music therapy on patients with UWS. In a pre-post method spanning 5 weeks, the neurological effect was measured by positron emission tomography, the vegetative effect by heart rate variability and behavioral changes by qualitative microanalysis of video sequences of the therapy sessions. Four patients were randomly enrolled either into the music therapy group (*n* = 2) or the control group (*n* = 2) by drawing lots. We want to illustrate two cases of patients in the music therapy group for this article to describe the neuroanthropological approach in a concrete example.

PET results of these two patients show that the brain activity during music therapy increased after 5 weeks of music therapy in three observed regions, which are the frontal areas (31/47%), the hippocampus (28% both) and the cerebellum (31/47%). These results show that the increase is quite high in both patients. Considering these results, we could assume that patients may have shown similar conditions too. But in fact they were different. Each patient has to be seen as a unique human being with their own resources and requirements for an individual path to recovery. This also means that the outcome of music therapy is often subjective and difficult to generalize. Therefore, we want to give a description of two of our patients, their music therapy sessions and the respective outcomes.

The first patient was a 70 year old man, who had been living with UWS after cardiac arrest for 3 years. His wife came to visit him at least every second day and provided a lot of information about his musical biography, preferences and interpretation of his physical conditions. When she saw the harp that the therapist brought for the first session, she mentioned that harp music had always been one of their favorites ever since they had had a wonderful vacation in Ireland with lots of happy memories and had wanted to go back there. Therefore, the therapist often worked with the harp during the first few weeks and played to him and also with him by guiding his hands and fingers through the strings. As the patient seemed to be more attentive in the course of the weeks, the therapist adapted the music to another piece of biographical information by singing songs of the 1950s, which the patient used to play in a band in his youth. In addition to singing the songs to the patient, the therapist guided his hands in the rhythm of the songs on a frame drum. The tempo and dynamics were adapted to the rhythm and condition of the patient, like, for example, breath and movement. All steps were described verbally to him.

The second patient was a 60 year old woman. She had been living with UWS for 4 years. In the first few sessions the patient experienced a high physical tension, had problems with secretion and seemed to be too stressed to listen or react to the music therapy. In a conversation with the patient's daughter the therapist found out that she liked to sing, especially traditional folk songs. As it was Christmas time, the therapist took up this information and sang Christmas and winter songs to her in a calm and rhythmic way and reduced verbal speech to a minimum. This was meant to give her the opportunity to calm herself down and regulate her physical tension. After rhythmicity came back into her breathing, the songs were often adjusted to this rhythm and included deep breaths or pauses.

The therapist constantly observed the patients attentively and tailored the therapy as well as the research setting to the present condition and needs of the patient. The therapy was, for example, adapted to the posture of the patient and the intensity of the light in the room was changed to fit the patient's individual sensitivity. The interpretation of the patient's needs, conditions and sensitivity was based on signs like facial expression, tonicity or breath. By acting attentively and describing everything that happened to or around the patients, the therapist guided them through the whole research process turning it into a reciprocal act.

After 5 weeks of music therapy various changes in the condition of both participants were observed in video micro analysis using the transcription software “Feldpartitur,” which allows an examination of video data by creating a score with various self-selected levels in intervals of 1 s. In a master's thesis these observed levels such as eyes, viewing direction, breathing of the patient and therapeutic intervention were evaluated in outcome categories (e.g., wakefulness, attention, tonus, communication) to gain insight into the behavioral changes after 5 weeks of music therapy. However, these changes differed in the two patients. While the subsequent video analysis indicated that the first patient's eyes were more open in the fifth week and he seemed to be more attentive, the second patient had her eyes closed most of the time and showed less movement. One could assume that she fell into a deeper state of the UWS. However, this is a rather limited perspective on a patient. Due to the individuality of the two patients and the contact in therapy, the changes are not comparable. From the beginning, the focus of the therapy was influenced by the behavior and condition of the patients and the intention of therapy was adapted to their capabilities and resources. Hence, the therapy with the first patient followed a more active approach and embraced his resources by engaging his wakefulness and attention. Due to the high tension of the second participant and her problems with secretion, an active approach would not have been appropriate and possibly even an excessive demand. The goal of music therapy for the second patient was to support her responses of relaxation and regulation.

The results show a positive outcome of music therapy with both patients. Music therapy helped the first patient to increase his wakefulness and attention. Several levels of observation in the video analysis represent this outcome: his eyes are more open in the fifth week, he looks at the instruments and his eyebrows are raised, indicating concentration. His breath, which had a constant rhythm in the first week, varies depending on the activity or intervention in the therapy. Guiding his hands on a drum or the harp helped him increase his attention and focus on the instruments. Therefore, therapy changed from a rather receptive approach to active interventions. Also, more interaction between the patient and the therapist can be observed in the video analysis. This is shown by an increase in verbal explanations and requests by the therapist as well as in the increase of attention by the patient himself. For the wife of the first patient this increase in attention was recognizable as well. She described that her husband started to look around, that more eye contact was possible and she felt that the music made him more alert than just talking to him. She also made use of the possibility to be around during the PET measurement sessions, where she was allowed to be in the same room as the radial assistants which had a very calming effect on her, being close to her husband.

The goal to support relaxation and regulation was also met for the second patient as the tonus in her body and her face decreased. The video analysis showed that in the fifth week secretion and stertorous breathing started to subside shortly after the beginning of the music therapy and ended completely after a few minutes. Her breathing even became constant and rhythmic again. At first glance her wakefulness and attention appeared to be reduced, having her eyes closed, lying relaxed and without movement. However, we interpreted the changes in her condition as positive, as she seemed to be more relaxed and physically stable. But how do we know if this is really true? Our interpretation does not only derive from video analysis, which poses as a snap-shot of single therapy sessions, but also from interdisciplinary exchanges within our research group, with care givers, relatives, therapists and neurologists and from the perception of the therapist during the therapy sessions. An important perspective and source of knowledge is the view of the patients' relatives, since they are usually very close to them and can provide valuable information about their behavioral change.

All of this information was recorded in protocols and a research diary which supported the analysis with qualitative data. According to Csordas (2004), “*Perception* becomes *data* when it is used as *evidence* to establish *facts*, which are subsequently elevated to the status of *truths* and *certainties*.” Subjective perception can be valuable and fruitful for research, especially if patients are not able to communicate their perception directly. Using it in a reflective process, it complements data by conveying the individual environment of a human being.

Therefore, we argue that important knowledge is not only generated via an objective machine but also via human interaction throughout the whole research process and in the therapeutic setting at the bedside. Whenever individuals come together, experience and knowledge are exchanged, which in turn creates new kinds of knowledge (Roepstorff, 2001).

However, working in an interdisciplinary team can also lead to difficulties, since people do not always share the same research background and framework of thoughts. Coming from different traditions, they are facing a shared process of knowledge creation (Roepstorff, 2001) and need to find a way to attune. Being confronted with the common tensions between ideals of methods and the realities of research (Roepstorff and Frith, 2012), we tried to identify these tensions and reflect on them. From a neuroanthropological perspective the aim is not to avoid these tensions but to be aware of them and to discuss their influence on the research results. Moreover, conflicts and tensions can bear chances of creativity, as they provide room for improvement. Tensions can be valuable, as they lead to a dialog between different professions and help consider a broader view.

The benefit of neuroanthropology lies in the open approach to the complexity of music therapy. The multidisciplinary proceedings in research allow us to capture various different processes within this complexity. But still, as in music therapy practice, the human individual is at the center of the whole research process. Additionally, it allows the acknowledgment of the qualitative value of music therapy.

## Conclusion

Neuroscience supports the understanding of the effect of music therapy at the level of brain activity of people with DOC. However, brain images, like PET, cannot sufficiently determine the *local world* and the impact on the patients' life. An additional behavioral observation, e.g., video analysis, can give information on a patient's condition. However, it is static and inflexible as it captures only a short period of time. As a patient with DOC is not able to express himself with the familiar means of communication, both neuroscience and behavioral observations pose a risk of misinterpretation. Neuroanthropology can help bridge this gap by reflecting on the information collected in the environment of the patient, which is necessary for a careful interpretation of data.

We suggest taking the entire research project including the experiences, sensation, meaning, and perception into consideration as an important source of knowledge, to bring together the different inner and outer worlds of every individual involved and to realize the importance, not only of the objective data but also of the lived experience, relationship aspects and all the social interactions.

When dealing with humans, and especially patients with DOC, the question should be how we can improve their situation. Is it the ideal to have tightly controlled experiments and behave like a personalized robot in order to collect “objective” data or could it be different? Could the research process itself be more open, fluent and flexible according to the individual and the situational needs? Another question that still needs consideration is: How would we then deal with the amount of data collected and measure a successful research result?

We know that in reality a research process can never be 100% controllable (Roepstorff, 2001). Why not take this finding and use it for research itself?

Combining anthropological methods like the ethnographic investigation with neuroscientific quantitative data collection and behavioral measures can provide further understanding of what is really happening during the music therapy interventions. In particular ethnographic insights can help us with the interpretation of research results and inspire us to ask novel questions (Domínguez et al., 2009).

The fact that an observed and described phenomenon is difficult to confirm does not provide sufficient evidence against its authenticity or importance. Nevertheless, we need to develop more powerful methods with which to study such phenomenon and thus make them comprehensible and valuable for a music therapist in daily clinical practice. DOC pose many unanswered questions due to the inability of the patients to communicate their own perceptions and views. However, considering not only snap-reading methods but a broader view of the patients' environment could help us gain more knowledge on music therapy and approach the patients directly.

To achieve this, an intense self-reflecting process of the therapist and researcher is required. The University of Applied Sciences Krems encourages and supports this self-reflecting process from the beginning of its music therapy program in order to impart the importance of a neuroanthropological perspective to future generations of music therapists and researches. This will hopefully help improve the neuroscientific research on music therapy and lead us toward a deeper comprehension of our work with the patients.

### Conflict of interest statement

The authors declare that the research was conducted in the absence of any commercial or financial relationships that could be construed as a potential conflict of interest.
